# Improved Visualization and Specific Binding for Metabotropic Glutamate Receptor Subtype 1 (mGluR1) Using [^11^C]ITMM with Ultra-High Specific Activity in Small-Animal PET

**DOI:** 10.1371/journal.pone.0130006

**Published:** 2015-06-15

**Authors:** Tomoteru Yamasaki, Masayuki Fujinaga, Joji Yui, Hidekatsu Wakizaka, Tomoyuki Ohya, Nobuki Nengaki, Masanao Ogawa, Yoko Ikoma, Akiko Hatori, Lin Xie, Kazunori Kawamura, Ming-Rong Zhang

**Affiliations:** Molucular Probe Program, Molecular Imaging Center, National Institute of Radiological Sciences, Chiba, Japan; Rutgers University, UNITED STATES

## Abstract

Metabotropic glutamate receptor subtype 1 (mGluR1) is a crucial target in the development of new medications to treat central nervous system (CNS) disorders. Recently, we developed *N*-[4-[6-(isopropylamino)pyrimidin-4-yl]-1,3-thiazol-2-yl]-4-[^11^C]methoxy-*N*-methyl-benzamide ([^11^C]ITMM) as a useful positron emission tomography (PET) probe for mGluR1 in clinical studies. Here, we aimed to improve visualization and threshold of specific binding for mGluR1 using [^11^C]ITMM with ultra-high specific activity (SA) of > 3,500 GBq/μmol in rat brains. A two-tissue compartment model indicated large differences between the two SAs in the constants *k_3_* and *k_4_*, representing binding ability for mGluR1, while constants *K*_*1*_ and *k_2_* showed no differences. The total distribution volume (*V_T_*) values of conventional and ultra-high SA were 9.1 and 11.2 in the thalamus, 7.7 and 9.7 in the striatum, and 6.4 and 8.5 mL/cm^3^ in the substantia nigra, respectively. The specific binding of [^11^C]ITMM with ultra-high SA was significantly higher than the conventional SA, especially in the basal ganglia. Parametric PET images scaled with *V_T_* of the ultra-high SA clearly identified regional differences in the rat brain. In conclusion, PET studies using [^11^C]ITMM with ultra-high SA could sufficiently improve visualization and specific binding for mGluR1, which could help further understanding for mGluR1 functions in CNS disorders.

## Introduction

Glutamate is a major neurotransmitter that triggers excitatory neurotransmission via receptor binding in the central nervous system (CNS). Two types of glutamate receptors are recognized: ionotropic and metabotropic. Metabotropic glutamate receptors (mGluRs) are G-protein-coupled receptors. They can be classified into three groups comprising eight subtypes, based on sequence homology, intracellular transduction pathways, and pharmacological properties [1, 2]. The mGluR subtype 1 (mGluR1) categorized in group I is coupled with Gq proteins and can stimulate polyphosphoinositide hydrolysis, resulting in the formation of the second messengers inositol 1, 4, 5-triphosphate and diacylglycerol. Subsequently, these messengers trigger intracellular calcium release and activation of protein kinase C [3–5].

Based on these biological properties, several antagonists for mGluR1 have been developed for pharmacological studies of CNS disorders [6–10]. Additionally, several radioactive probes for mGluR1 for use in positron emission tomography (PET) have been developed to investigate mGluR1 function [11–15]. For example, the recently developed *N*-[4-[6-(isopropylamino)pyrimidin-4-yl]-1,3-thiazol-2-yl]-4-[^11^C]-methoxy-*N*-methyl-benzamide ([^11^C]ITMM) has initially been used for PET studies in animals and has exhibited high affinity (Ki = 12.6 nM) for mGluR1 [16]. [^11^C]ITMM is derived from 4-fluoro-*N*-[4-[6-(isopropylamino)pyrimidin-4-yl]-1,3-thiazol-2-yl]-*N*-methylbenzamide (FITM), a potent negative allosteric modulator of mGluR1 [10]. Subsequently, [^11^C]ITMM has been translated to first-in-human PET studies [17, 18]. In PET study of human brain, the total distribution volume (*V*_*T*_) of [^11^C]ITMM was reported as 2.61 mL/cm^3^ for the cerebellum (mGluR1-rich region) and 0.52 mL/cm^3^ for the pons (mGluR1-negligible region). The ranked order of corresponding *V*_*T*_ of [^11^C]ITMM was cerebellum > thalamus > frontal cortex > striatum ≈ pons [18]. This distribution pattern was consistent with the known biological distribution of mGluR1 in primate brains [13].

To our knowledge, [^11^C]ITMM is the only PET probe used to visualize mGluR1 that has been implemented in human clinical studies. Recently, several selective modulators have been used in clinical studies. The pharmacological properties of these drugs may indicate their use as treatments for psychiatric illnesses in which there is dysfunction of neuronal networks of the basal ganglia, such as in anxiety, depression, and schizophrenia [19]. PET imaging of mGluR1 in the basal ganglia is helpful for elucidating interactions between the expression of mGluR1 and disorders of these neuronal networks. However, radioactive uptake of [^11^C]ITMM in the human striatum is low, even though mGluR1 is present throughout the mammalian brain, including the basal ganglia [2]. Therefore, to apply findings from PET (including imaging, quantification and drug occupancy assessments) to the study of these disorders, the threshold of specific binding of [^11^C]ITMM for mGluR1 in the basal ganglia should be expanded as possible. The specific binding of radioprobe can be increased in two ways:

1. development of a radioprobe with higher binding affinity for the target molecule;
2. reduction of contamination by the nonradioactive carrier using radioprobe with high SA.

In the current study, we performed PET imaging using [^11^C]ITMM with ultra-high SA (3,515–9,620 GBq/μmol) to improve visualization and specific binding for mGluR1.

## Materials and Methods

### General

All experiments were approved by the committee of the National Institute of Radiological Sciences. All chemicals and solvents used in this study were of analytic or high-performance liquid chromatography (HPLC) grade and purchased from Sigma-Aldrich (St. Louis, MO, USA), Wako Pure Industries (Osaka, Japan), and Tokyo Chemical Industries (Tokyo, Japan).

Male Sprague-Dawley (SD) rats were purchased from Japan SLC (Shizuoka, Japan), kept under the temperature-controlled environment with a 12-h light-dark cycle, and fed a standard diet (MB-1; Funabashi Farm, Chiba, Japan).

### Ethics statement

The rats were treated and handled according to the Recommendations for Handling of Laboratory Animals for Biomedical Research, compiled by the Committee on the Safety and Ethical Handling Regulations for Laboratory Animal Experiments, National Institute of Radiological Sciences, and this study was approved by the committee.

### Radiochemistry

**Fig 1:**
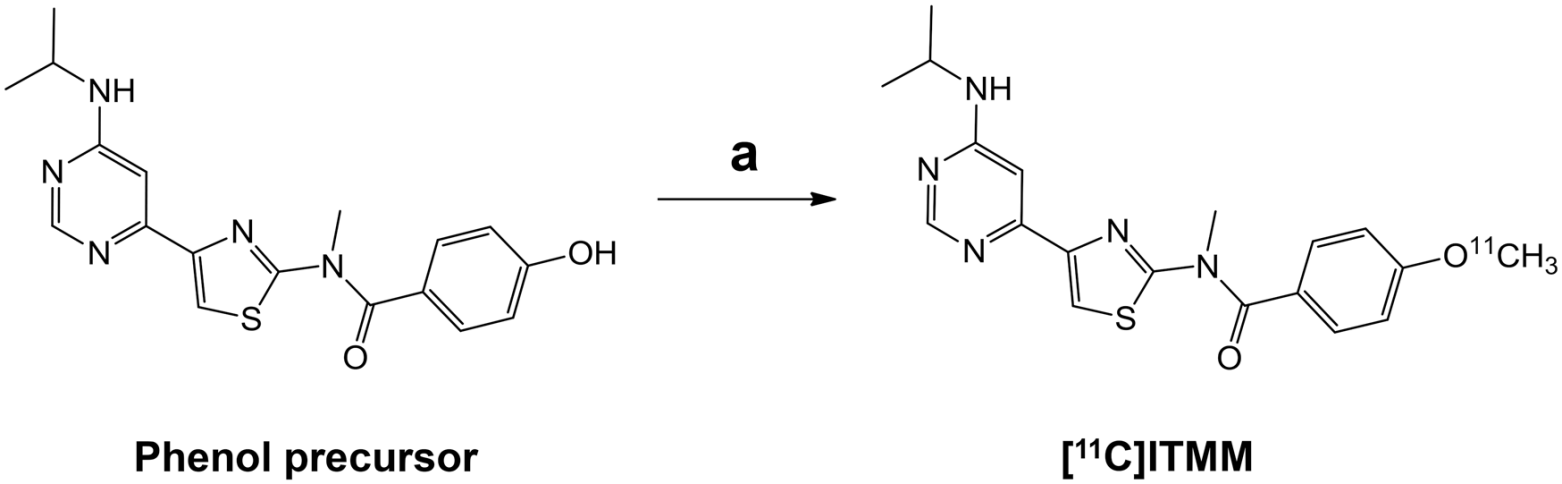
Radiosynthesis of [^11^C]ITMM with conventional or ultra-high SA.

[^11^C]ITMM, with either conventional or ultra-high SA, was synthesized as described previously [16]. Briefly, for the conventional SA, [^11^C]CH_3_I was prepared from cyclotron-produced [^11^C]CO_2_ [20]. For the ultra-high SA, [^11^C]CH_3_I was produced by iodination of [^11^C]CH_4_ formed in the target chamber in situ (the single pass I_2_ method) [21]. [^11^C]ITMM with conventional or ultra-high SA was obtained by reacting the phenol precursor with [^11^C]CH_3_I in *N*,*N*-dimethylformamide (DMF) at 70°C for 5 min (Fig 1).

For the current study, [^11^C]ITMM with conventional SA was synthesized with 27 ± 5% radiochemical yield (decay-corrected). Starting from 14–20 GBq of [^11^C]CO_2_, 1.4–2.8 GBq of [^11^C]ITMM was produced 27 ± 3 min after the end of bombardment (EOB). The radiochemical purity was higher than 99% and 121 ± 17 GBq/μmol (range: 74–174 GBq/μmol) of SA was obtained at the end of synthesis (EOS). [^11^C]ITMM with ultra-high SA was synthesized with 6 ± 1% radiochemical yield (decay-corrected). Starting from approximately 37 GBq of [^11^C]CH_4_, 0.7–1.5 GBq of [^11^C]ITMM was produced within 32 min from EOB. The radiochemical purity was higher than 99% and 5,794 ± 1,022 GBq/μmol (range: 3,515–9,620 GBq/μmol) of SA was obtained at the EOS.

### PET procedure with blood sampling

Prior to PET assessment, a SD rat had a polyethylene catheter (FR2, Imamura, Tokyo, Japan) inserted into the left femoral artery for blood sampling. Subsequently, a SD rat was secured in a custom-designed chamber and placed in a small-animal PET scanner (Inveon, Siemens Medical Solutions, Knoxville, TN, USA). Body temperature was maintained using a 40°C water circulation system (T/Pump TP401, Gaymar Industries, Orchard Park, NY, USA). A 24-gauge intravenous catheter (Terumo Medical Products, Tokyo, Japan) was placed in the tail vein of the rat for a bolus injection.

A bolus (0.5 mL) of [^11^C]ITMM with conventional or ultra-high SA was injected for 10 s via a catheter inserted in the tail vein. Dynamic emission scans in three-dimensional list mode were performed for 90 min (10 s x 12 frames, 20 s x 3 frames, 30 s x 3 frames, 60 s x 3 frames, 150 s x 3 frames, and 300 s x 15 frames). For counting radioactivity, blood samples were manually acquired at intervals of 10 s (0.05 mL for 50 s), 1-min intervals (0.05 mL for 5 min), and then at 10 (0.05 mL), 15 (0.2 mL), 30 (0.3 mL), 60 (0.4 mL), and 90 min (0.5 mL) after the PET scan started. For metabolite analysis, six samples of blood were obtained at 1 (0.02 mL), 5 (0.02 mL), 15 (0.05 mL), 30 (0.1 mL), 60 (0.2 mL), and 90 min (0.3 mL) after the injection. Blood samples were centrifuged at 13,000*g* at 4°C to separate the plasma. Levels of radioactivity in the whole blood and plasma were counted using a 1480 Wizard auto-gamma scintillation counter (Perkin-Elmer, Waltham, MA, USA). The radioactivity was corrected for decay.

Metabolite analysis was performed as described previously [22]. Briefly, whole blood samples were treated to separate the plasma, which was deproteinized with an equivalent amount of acetonitrile. An aliquot of the supernatant obtained from the plasma was analyzed by HPLC with a radiation detector [23]. Plasma protein binding was not determined in the present study. The time curves for the fraction of unchanged [^11^C]ITMM in the plasma were fitted using three exponential equations, and subsequently used for kinetic analysis.

PET dynamic images were reconstructed with filtered back projection using a Hunning filter with a Nyquist cut-off of 0.5 cycle/pixel. PET/MRI fused images were acquired by PMOD version 3.4 (PMOD Technologies, Zurich, Switzerland). To generate the time-activity curves (TACs) of specified brain regions, volumes of interest (VOIs) were drawn encompassing the cerebellum, hippocampus, thalamus, striatum, cingulate cortex, substantia nigra, and pons. The radioactivity was decay-corrected to the injection time and is expressed as the standardized uptake value (SUV), which was normalized to the injected radioactivity and body weight [24]. SUV was calculated according to the following formula: SUV = (radioactivity per milliliter tissue / injected radioactivity) × gram body weight.

### Estimation of equilibrium state

Estimations of the equilibrium state of [^11^C]ITMM in each brain region were obtained using averaged plasma input functions and averaged tissue TACs. Theoretical radioactive concentrations in each brain region (*C*_*t*_ *’*) and the plasma input function of [^11^C]ITMM (*C*_*P*_ *’*) were estimated using averaged measurements extracted between 5 and 90 min after the injection and subjected to three exponential least square fitting [Radioactivity (t) = A*exp(-at) + B*exp(-bt) + C*exp(-ct)].

The estimations over 90 min after the injection were calculated by extrapolating based on the equation as described above. The time at which equilibrium state was reached in each brain region was determined by the peak of the time-course of tissue-to-plasma concentration ratios, *C*_*t*_ *’*(t)/*C*_*P*_ *’*(t).

To confirm validity of PET scanning time in this study, scatter plot analysis of measured and estimated values was performed using *V*_*T*_ values based on Logan graphical analysis (GA) [25].

### Two-tissue compartment model analysis

Kinetic analysis of [^11^C]ITMM was performed using the two-tissue compartment model (2TCM) [26, 27]. The compartments correspond to the radioactivity concentrations of unchanged radioprobe in the plasma (*C*_*P*_), free and non-specifically bound radioprobe (*C*_*1*_), and radioprobe specifically bound to receptors (*C*_*2*_). The rate constants *K*_*1*_ and *k*_*2*_ correspond to the influx and outflux rates of the radioprobe across the blood-brain barrier. The rate constants *k*_*3*_ and *k*_*4*_ correspond to the rates of radioprobe transfer between the *C*_*1*_ and *C*_*2*_ compartments. The blood volume fraction (*vB*) in the rat brain was fixed at 2% [28]. The four rate constants of the 2TCM were obtained by a nonlinear least squares fitting technique, using PMOD version 3.4 (PMOD Technologies).

The distribution of [^11^C]ITMM in free or tissue compartments was described using the concept of free ligand distribution volume (*DV*_*f*_) or *V*_*T*_, which was defined by the following equations:

$$DV_{f}=\frac{K_{1}}{k_{2}}$$ (1)

$$V_{T}=DV_{f}\left(1+\frac{k_{3}}{k_{4}}\right)$$ (2)

The nondisplacable binding potential (*BP*_*ND*_) is defined using the index of specific bindings of the radioprobe, meaning the receptor density (*B*_*max*_) to binding affinity (*K*_*D*_) ratio, which corresponds with the ratio of *k*_*3*_ to *k*_*4*_ in the 2TCM [26]. The *BP*_*ND*_ is defined as follows:

$$BP_{ND}=f_{ND}\cdot \frac{B_{\text{max}}}{K_{D}}=\frac{k_{3}}{k_{4}}$$ (3)

Here, *f*_*ND*_ represents the tissue-free fraction of radioprobe in the nondisplacable compartment [29]. Although the fraction of radioprobe that is unbound to plasma proteins was not measured in the present study, the value of *f*_*ND*_ is close to 1.0 [29].

### Parametric images

The ratio of vascular radioactivity contribution was fixed at 2%, and eliminated prior to the parameter estimation. Parameter estimates were considered outliers if *V*_*T*_ value was outside the range 0 < *V*_*T*_ < 25. The parametric image was acquired in PMOD version 3.4 (PMOD Technologies).

### Statistics

Kinetic model selection was determined using three statistical methods: the Akaike information criterion [30]; the Schwarz criterion [31]; and the Model Selection criterion.

The standard error (s.e.) of the parameter was given by the diagonal of the covariance matrix, expressed as a percentage of the parameter value (coefficient of variation, %COV) and used to validate the parameter by a nonlinear least square fitting procedure [32].

All data are expressed as the mean ± s.e. Differences between the two SAs were calculated using a two-way ANOVA. Significance was determined at p < 0.05. The data were analyzed by GraphPad Prism 5 software (GraphPad Software, San Diego, CA, USA).

## Results

### Small-animal PET study

[^11^C]ITMM with ultra-high SA of 3,515–9,620 GBq/μmol contained 10.9 ± 1.6 pmol of nonradioactive carrier in the injected solution. Meanwhile, [^11^C]ITMM conventional SA of 74–174 GBq/μmol included 513.1 ± 58.7 pmol of nonradioactive carrier in the injected solution (Table 1).

**Table 1:** Characteristics of animals used and experimental data from the two conditions using [^11^C]ITMM with conventional or ultra-high SA in PET (means ± s.e.).

	Conventional SA	Ultra-high SA
Age (weeks old)	7–9	8
Gender	male	male
Body weight (g)	280.9 ± 10.1	281.2 ± 6.7
Subjects	5	5
Radioactivity (MBq)/head	57.7 ± 1.2	56.6 ± 1.4
Carrier dose (pmol)/head	513.1 ± 58.7	10.9 ± 1.6

**Fig 2:**
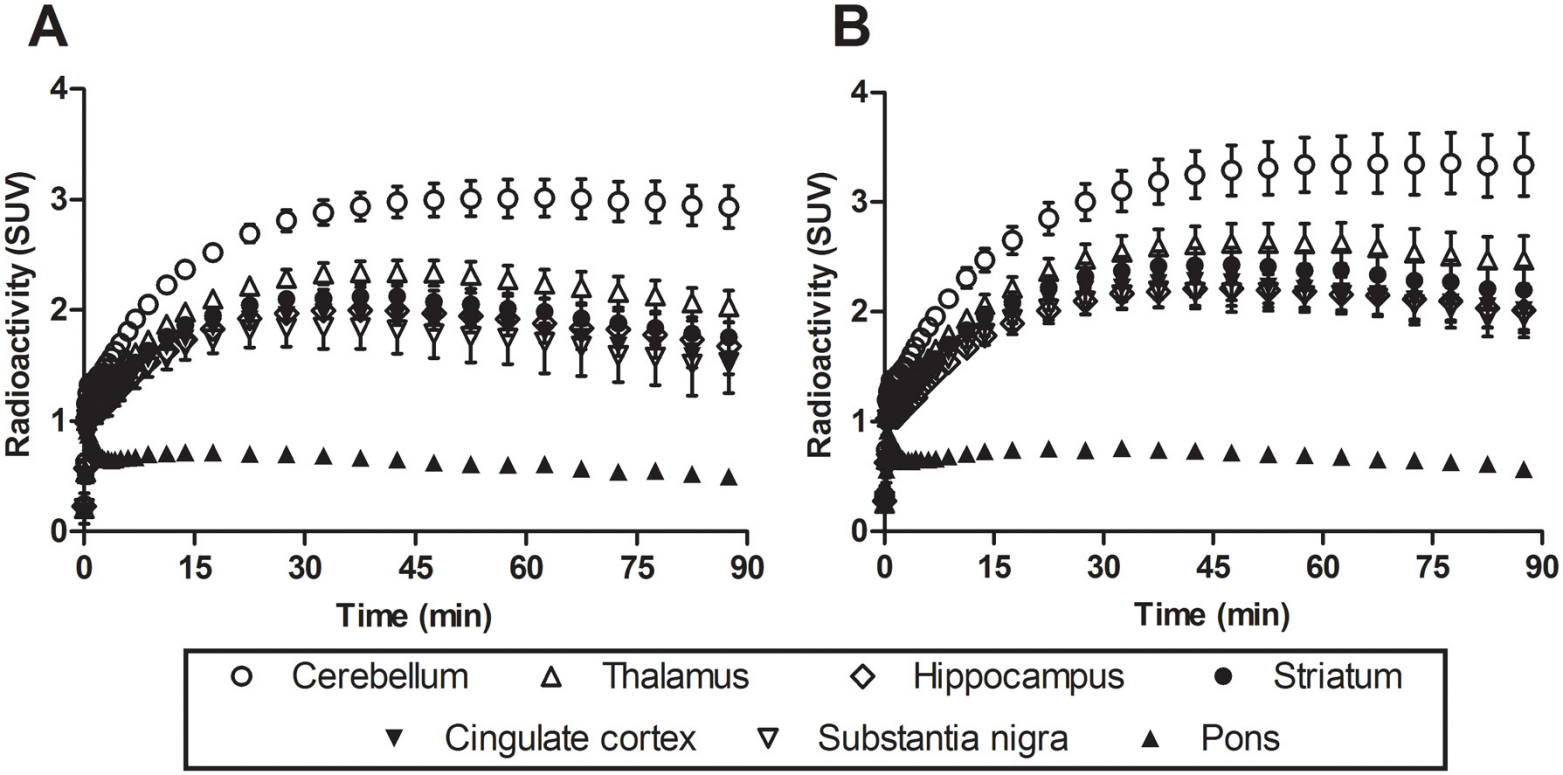
Time-activity curves of [^11^C]ITMM with (A) conventional SA and (B) ultra-high SA.

Fig 2 shows TACs of [^11^C]ITMM with conventional (A) or ultra-high (B) SA in the cerebellum, thalamus, hippocampus, striatum, cingulate cortex, substantia nigra, and pons. The maximum uptake of [^11^C]ITMM with the conventional SA was 3.0 in the cerebellum, 2.3 in the thalamus, 2.0 in the hippocampus, 2.1 in the striatum, 2.0 in the cingulate cortex, 1.8 in the substantia nigra, and 0.9 SUV in the pons. Meanwhile, the maximum uptake of [^11^C]ITMM with ultra-high SA was 3.4 in the cerebellum, 2.6 in the thalamus, 2.2 in the hippocampus, 2.4 in the striatum, 2.3 in the cingulate cortex, 2.2 in the substantia nigra, and 0.9 SUV in the pons. Moreover, the peak time of radioactivity with the ultra-high SA was delayed by 4–10 min compared with the conventional SA.

### Estimation of equilibrium state

**Fig 3:**
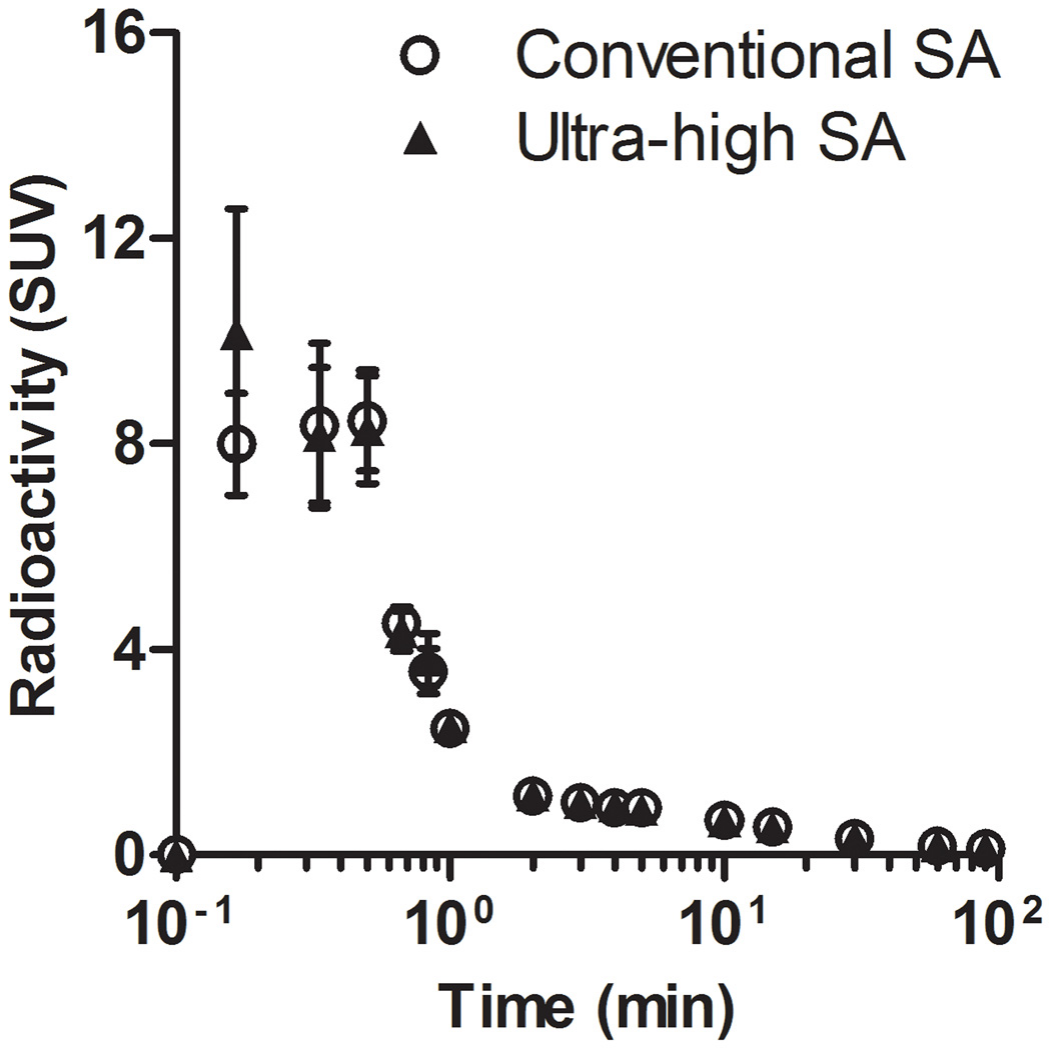
The metabolite-corrected input function of [^11^C]ITMM with conventional or ultra-high SA in the plasma.

The metabolite corrected input functions after intravenous injection of [^11^C]ITMM with conventional and ultra-high SA are shown in Fig 3. Radioactivity appeared rapidly, initially (< 1 min) at high levels in the plasma, and then it quickly decreased. The radioactivity in the plasma was roughly 55% of unchanged [^11^C]ITMM at 15 min, decreasing to roughly 15% 90 min after the injection of the two SAs (Table 2).

**Table 2:** Percentage of unchanged [^11^C]ITMM with conventional or ultra-high SA in the plasma (means ± s.e., n = 5 in each group).

Time after injection (min)	Unchanged form (%)	
	Conventional SA	Ultra-high SA
1	95.2 ± 1.2	97.3 ± 0.6
5	74.0 ± 2.1	77.1 ± 3.2
15	53.7 ± 2.6	56.2 ± 4.0
30	36.8 ± 2.6	40.0 ± 4.0
60	21.7 ± 2.0	24.7 ± 2.9
90	15.7 ± 1.3	18.2 ± 2.5

**Fig 4:**
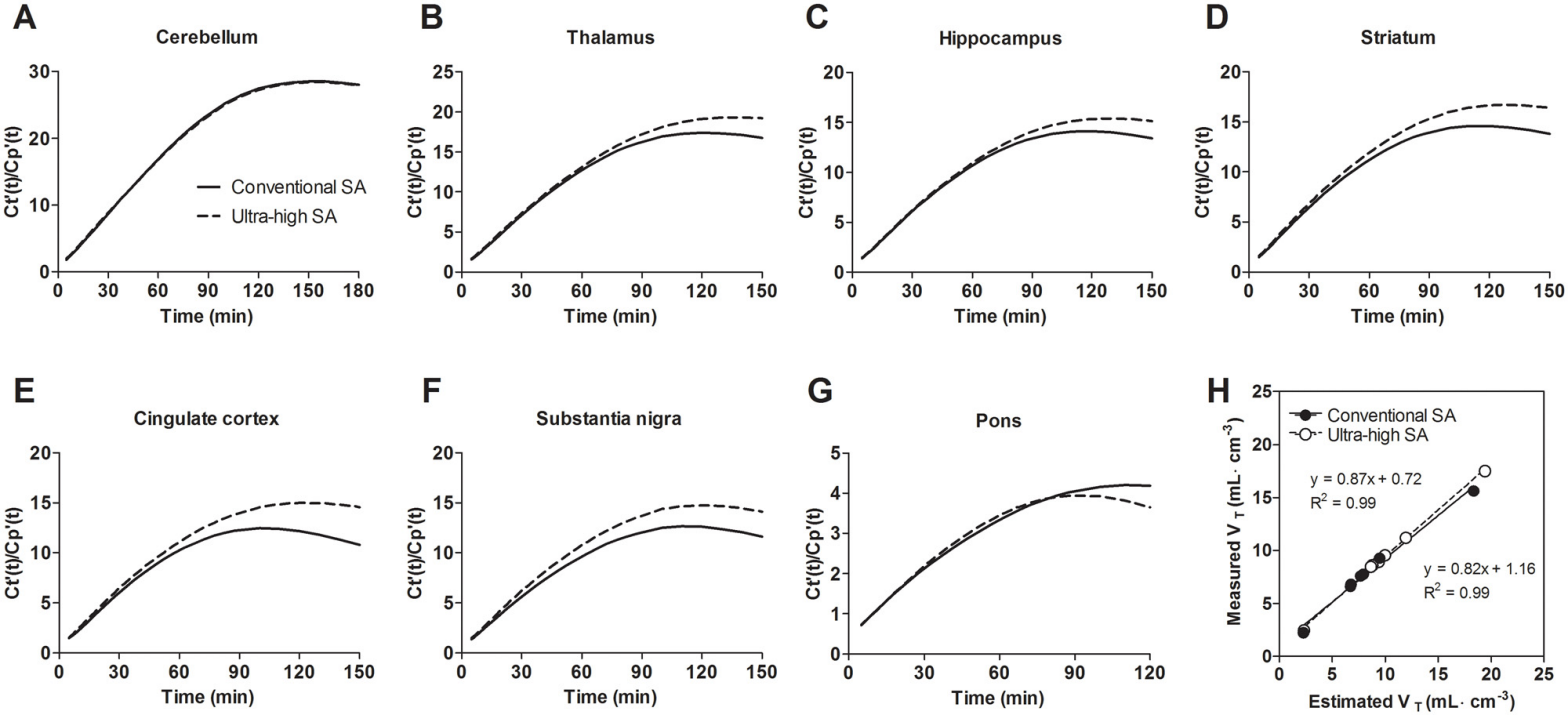
Estimated tissue-to-plasma concentration ratio, *C*_*t*_ *’*(t)/*C*_*P*_ *’*(t), of [^11^C]ITMM with conventional or ultra-high SA and scatter plots indicating the relationship between measured- and estimated-*V*_*T*_ values.

Fig 4 shows time-courses of estimated tissue-to-plasma concentration ratios, *C*_*t*_ *’*(t)/*C*_*P*_ *’*(t), for [^11^C]ITMM with conventional or ultra-high SA in the (A) cerebellum, (B) thalamus, (C) hippocampus, (D) striatum, (E) cingulate cortex, (F) substantia nigra, and (G) pons. The estimated equilibrium times (min) of [^11^C]ITMM with conventional and ultra-high SA were 150 and 150 for the cerebellum, 120 and 140 for the thalamus, 120 and 130 for the hippocampus, 120 and 130 for the striatum, 100 and 120 for the cingulate cortex, 110 and 100 for the substantia nigra, and 110 and 100 for the pons, respectively. In this study, PET assessments with blood sampling were performed for 90 min because of the short half-life of ^11^C. Uptake of radioactivity of [^11^C]ITMM with conventional and ultra-high SA in the brain regions reached 80 and 82 of equilibrium state in the cerebellum, 91 and 87 in the thalamus, 93 and 89 in the hippocampus, 93 and 90 in the striatum, 96 and 91 in the cingulate cortex, 94 and 98 in the substantia nigra, and 94 and 98% in the pons, respectively.

Fig 4H shows correlations between measured- and estimated-*V*_*T*_ values based on Logan GA. A high correlation was seen between the measured and estimated values.

### Two-tissue compartment model analysis

Details of full kinetic parameters using 2TCM with metabolite-corrected plasma input functions are given in Table 3 for conventional SA and in Table 4 for ultra-high SA. The rate constants (*K*_*1*_–*k*_*4*_) in both experimental groups were acquired with relatively low %COV. The range of %COV for constant *K*_*1*_ in conventional and ultra-high SA experiments was 3.5–9.9 and 2.1–9.1, respectively. For constant *k*_*2*_, it was 9.6–25.3 and 8.9–22.9. For constant *k*_*3*_, it was 6.5–14.4 and 4.3–12.6. For constant *k*_*4*_, it was 3.4–11.6 and 3.0–10.2.

**Table 3:** Full kinetic parameters of [^11^C]ITMM with conventional SA using 2TCM.

	Region	*K*_*1*_ (mL/cm^3^/min)	*k*_*2*_ (/min)	*k*_*3*_ (/min)	*k*_*4*_ (/min)	*V*_*T*_ (mL/cm^3^)	*DV*_*f*_ (*K*_*1*_/*k*_*2*_)	*BP*_*ND*_ (*k*_*3*_/*k*_*4*_)	AUC (SUV · min)
Mean ± s.e. (n = 5)									
	Cerebellum	0.344 ± 0.010	0.811 ± 0.045	0.619 ± 0.035	0.019 ± 0.001	14.56 ± 0.50	0.429 ± 0.020	33.2 ± 1.4	243.2 ± 9.2
	Thalamus	0.303 ± 0.012	0.886 ± 0.064	0.669 ± 0.033	0.026 ± 0.001	9.12 ± 0.37	0.347 ± 0.018	25.5 ± 1.2	188.4 ± 6.9
	Hippocampus	0.303 ± 0.015	1.052 ± 0.082	0.644 ± 0.051	0.026 ± 0.001	7.44 ± 0.27	0.292 ± 0.011	24.8 ± 1.8	160.8 ± 4.6
	Striatum	0.278 ± 0.014	0.769 ± 0.061	0.612 ± 0.036	0.031 ± 0.001	7.66 ± 0.28	0.367 ± 0.015	20.0 ± 1.1	169.7 ± 6.0
	Cingulate cortex	0.312 ± 0.008	1.062 ± 0.086	0.643 ± 0.053	0.030 ± 0.001	6.76 ± 0.25	0.302 ± 0.021	21.7 ± 1.2	156.9 ± 5.3
	Substantia nigra	0.298 ± 0.021	0.840 ± 0.128	0.470 ± 0.031	0.029 ± 0.001	6.44 ± 0.22	0.378 ± 0.030	16.6 ± 1.6	149.9 ± 6.7
	Pons	0.271 ± 0.015	1.237 ± 0.122	0.256 ± 0.022	0.031 ± 0.001	2.06 ± 0.06	0.224 ± 0.011	8.3 ± 0.5	56.0 ± 1.6
%COV									
	Cerebellum	5.90 ± 0.51	17.73 ± 0.89	10.56 ± 0.53	5.95 ± 0.34	2.53 ± 0.25	12.38 ± 0.53	12.3 ± 0.5	-
	Thalamus	5.46 ± 0.56	16.27 ± 1.16	9.79 ± 0.78	4.99 ± 0.37	1.65 ± 0.19	11.32 ± 0.79	11.5 ± 0.8	-
	Hippocampus	6.15 ± 0.41	16.30 ± 1.02	9.76 ± 0.78	5.05 ± 0.36	1.86 ± 0.17	10.78 ± 0.79	10.9 ± 0.8	-
	Striatum	5.23 ± 0.48	16.69 ± 1.50	10.69 ± 1.14	5.12 ± 0.48	1.58 ± 0.18	11.95 ± 1.22	12.3 ± 1.3	-
	Cingulate cortex	6.97 ± 0.72	18.28 ± 1.74	10.94 ± 0.82	5.52 ± 0.39	1.88 ± 0.10	12.01 ± 1.14	12.3 ± 1.2	-
	Substantia nigra	5.87 ± 0.41	15.36 ± 0.87	10.18 ± 1.23	5.37 ± 0.43	2.06 ± 0.18	10.10 ± 1.12	10.5 ± 1.2	-
	Pons	7.72 ± 1.07	13.32 ± 1.24	11.38 ± 0.86	8.95 ± 0.69	4.01 ± 0.29	6.83 ± 0.47	8.0 ± 0.6	-

*V*_*T*_: total distribution volume; *DV*_*f*_: distribution volume of free ligand; *BP*_*ND*_: nondisplacable binding potential; AUC: area under the curve.

**Table 4:** Full kinetic parameters of [^11^C]ITMM with ultra-high SA using 2TCM.

	Region	*K*_*1*_ (mL/cm^3^/min)	*k*_*2*_ (/min)	*k*_*3*_ (/min)	*k*_*4*_ (/min)	*V*_*T*_ (mL/cm^3^)	*DV*_*f*_ (*K*_*1*_/*k*_*2*_)	*BP*_*ND*_ (*k*_*3*_/*k*_*4*_)	AUC (SUV · min)
Mean ± s.e. (n = 5)									
	Cerebellum	0.366 ± 0.019	1.112 ± 0.098	0.799 ± 0.035	0.016 ± 0.000	16.69 ± 0.92	0.336 ± 0.020	48.9 ± 2.6	269.9 ± 15.8
	Thalamus	0.313 ± 0.016	1.125 ± 0.088	0.817 ± 0.025	0.021 ± 0.000	11.15 ± 0.51	0.284 ± 0.018	38.5 ± 1.1	214.2 ± 12.2
	Hippocampus	0.320 ± 0.020	1.415 ± 0.107	0.784 ± 0.027	0.021 ± 0.000	8.93 ± 0.47	0.228 ± 0.008	38.2 ± 1.5	180.2 ± 10.9
	Striatum	0.294 ± 0.014	0.972 ± 0.083	0.722 ± 0.043	0.024 ± 0.001	9.66 ± 0.41	0.311 ± 0.021	30.6 ± 1.8	197.3 ± 11.1
	Cingulate cortex	0.339 ± 0.019	1.317 ± 0.054	0.740 ± 0.036	0.023 ± 0.001	8.66 ± 0.45	0.257 ± 0.008	32.8 ± 1.9	184.7 ± 11.1
	Substantia nigra	0.292 ± 0.012	0.933 ± 0.075	0.602 ± 0.036	0.023 ± 0.001	8.53 ± 0.47	0.321 ± 0.023	26.1 ± 2.0	178.9 ± 10.8
	Pons	0.244 ± 0.004	1.131 ± 0.084	0.253 ± 0.011	0.026 ± 0.001	2.39 ± 0.23	0.221 ± 0.015	9.8 ± 0.5	62.7 ± 3.6
%COV									
	Cerebellum	4.38 ± 0.84	10.51 ± 2.64	6.54 ± 0.76	4.64 ± 0.22	1.99 ± 0.15	7.31 ± 1.46	7.5 ± 1.3	-
	Thalamus	5.27 ± 0.90	14.76 ± 1.91	8.07 ± 0.92	4.45 ± 0.52	1.52 ± 0.18	9.96 ± 1.13	9.9 ± 1.1	-
	Hippocampus	5.68 ± 0.80	13.46 ± 1.46	7.13 ± 0.69	3.98 ± 0.39	1.61 ± 0.17	8.34 ± 0.77	8.3 ± 0.8	-
	Striatum	4.55 ± 0.67	13.17 ± 1.68	7.60 ± 0.95	4.02 ± 0.50	1.39 ± 0.20	9.03 ± 1.09	9.1 ± 1.1	-
	Cingulate cortex	6.42 ± 0.56	15.82 ± 1.48	8.78 ± 0.84	4.77 ± 0.45	1.86 ± 0.16	10.05 ± 1.02	10.0 ± 1.0	-
	Substantia nigra	5.34 ± 0.46	14.62 ± 1.00	8.90 ± 0.67	4.73 ± 0.35	1.90 ± 0.17	9.82 ± 0.63	9.9 ± 0.6	-
	Pons	6.15 ± 0.54	11.45 ± 0.70	10.05 ± 0.63	8.22 ± 0.60	3.84 ± 0.25	6.39 ± 0.29	7.2 ± 0.3	-

**Fig 5:**
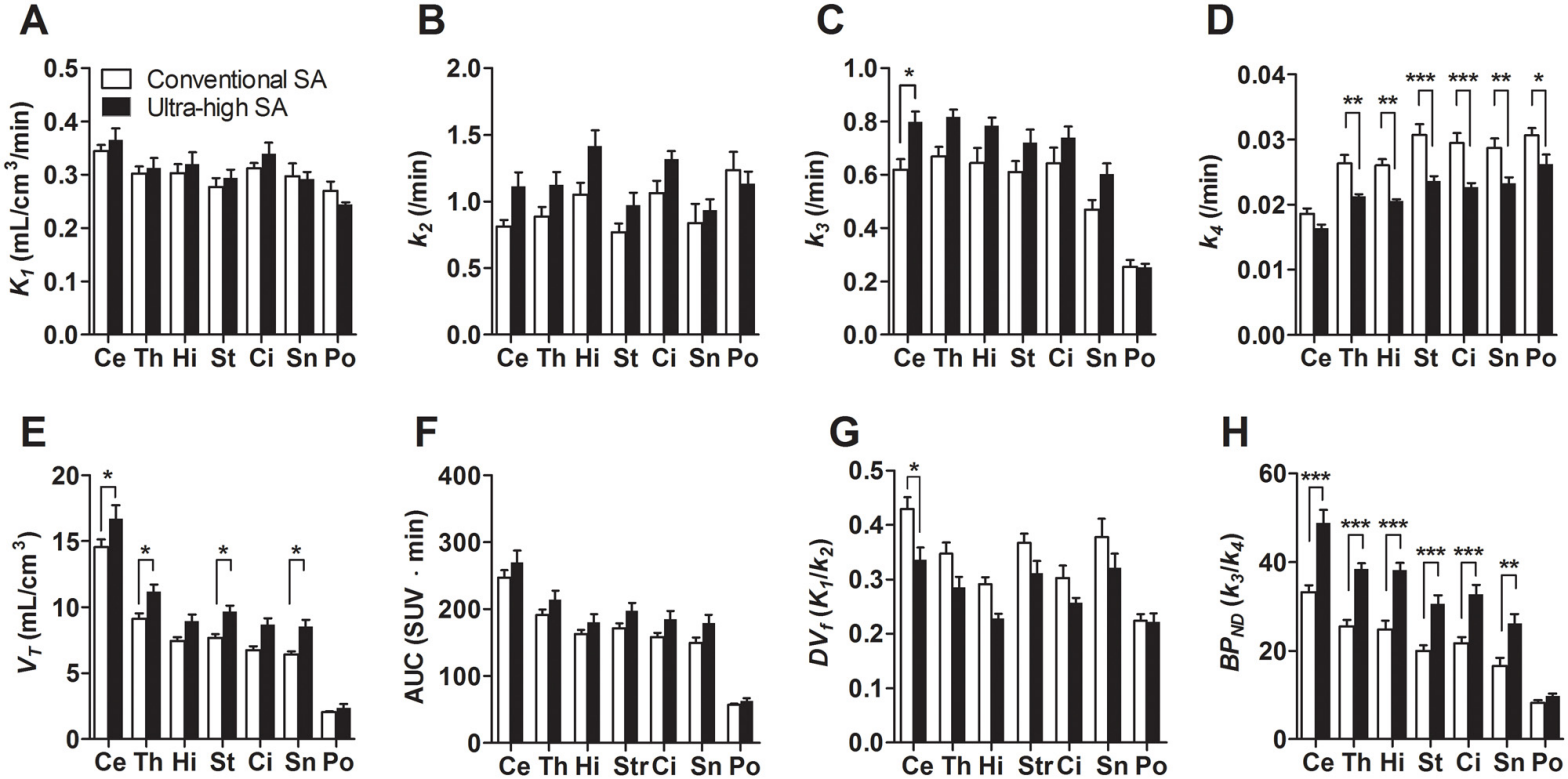
Differences in kinetic parameters between [^11^C]ITMM with conventional and ultra-high SA.

A comparison of kinetic parameters between the two SAs is shown in Fig 5. There were no differences in *K*_*1*_ between the two SAs in any brain regions. Meanwhile, *k*_*2*_ in the ultra-high SA tended to be higher compared with the conventional SA, except in the pons. Moreover, *k*_*3*_ in the ultra-high SA was higher in all brain regions except the pons compared with the conventional SA. In contrast, *k*_*4*_ of the thalamus, hippocampus, striatum, cingulate cortex, substantia nigra, and pons in the ultra-high SA condition was significantly lower compared with the conventional SA. Correspondingly, the *V*_*T*_, area under the curve (AUC) and *BP*_*ND*_ in the ultra-high SA condition were higher than in the conventional SA. In particular, significant differences (p < 0.001) were observed in the *BP*_*ND*_ of the two SAs in the cerebellum, thalamus, hippocampus, striatum, cingulate cortex, and substantia nigra. Conversely, the *DV*_*f*_ of each brain region in the ultra-high SA condition tended to be lower compared with the conventional SA condition.

### Parametric PET imaging

**Fig 6:**
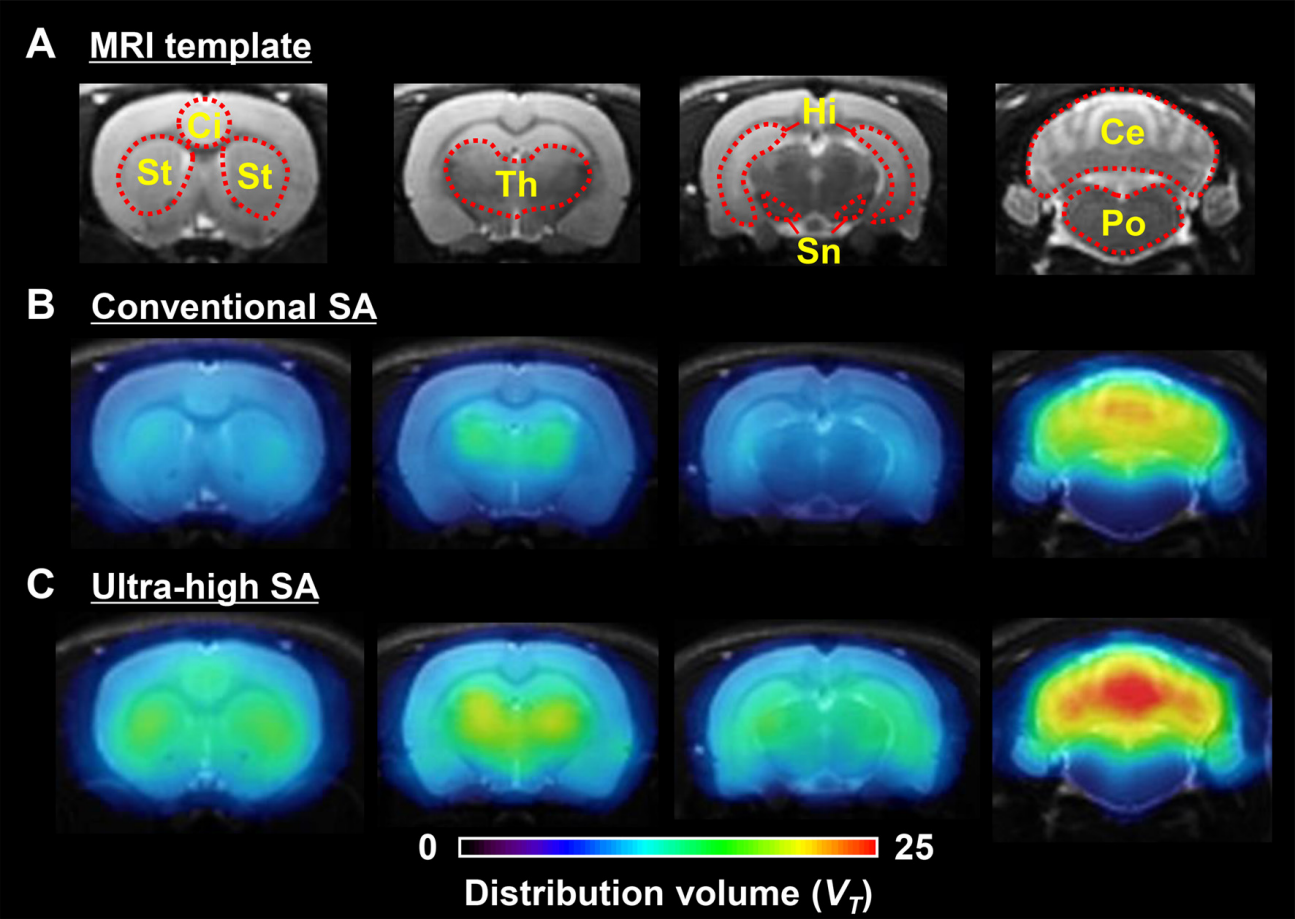
Representative parametric PET/MRI fused images based on *V*_*T*_ scale.

Fig 6 shows representative parametric PET/MRI fused images scaled according to *V*_*T*_. The signals for [^11^C]ITMM with ultra-high SA were noticeably higher than with the conventional SA in the cingulate cortex, striatum, thalamus, hippocampus, substantia nigra, and cerebellum. In particular, signals of the cingulate cortex and substantia nigra in the ultra-high SA were more identifiable than that in the conventional SA.

## Discussion

In the present study, we demonstrated that the specific binding of [^11^C]ITMM with ultra-high SA for mGluR1 was significantly increased in several brain regions compared with that of [^11^C]ITMM with conventional SA. In addition, *V*_*T*_-scaled parametric PET images of [^11^C]ITMM with ultra-high SA allowed more accurate identification of regional differences in mGluR1 than with the conventional SA.

Several [^11^C]radioprobes have been developed with ultra-high SA to increase specific binding and to visualize target molecules with low density [21, 33–37]. In the case of [^11^C]DAC, a selective PET probe for a translocater protein (TSPO) known to be a biomarker of inflammation, ultra-high SA has been demonstrated to exhibit a binding potential on the ipsilateral side of an ischemic rat brain roughly two times higher than with the conventional SA [36]. Thus, use of radioprobes with ultra-high SA has the potential to improve visualization and threshold of specific binding for target molecules with low density.

[^11^C]ITMM with ultra-high SA has been successfully synthesized as reported previously, but is yet to be evaluated as its application has not been investigated [16]. Here, the comparison between [^11^C]ITMM with conventional and ultra-high SA was performed using kinetic analysis by 2TCM.

Averaged PET images of [^11^C]ITMM with ultra-high SA showed a slight improvement compared with the conventional SA (S1 Fig). Additionally, its TAC indicated increased radioactive uptake in several brain regions (Fig 2). However, the ratio of tissue-to-plasma concentrations of [^11^C]ITMM did not reach equilibrium in either SA condition during the 90-min acquisition. Prior to kinetic analysis with 2TCM, we estimated equilibrium states by simulating estimated plasma input functions and tissue TACs (S2 and S3 Figs). The equilibrium times in both SA conditions were determined within 100–150 min after the injection (Fig 4A–4G). Unfortunately, PET with [^11^C]ITMM was impossible to perform under the equilibrium state, because radioactivity of plasma input function 90 min after the injection was very low. More importantly, [^11^C]ITMM bindings reached 80–98% of the equilibrium state, and there was high correlation between measured and estimated *V*_*T*_ values in both SA conditions (Fig 4H). These results permit kinetic analysis with 2TCM using tissue TACs acquired from PET scans of 90 min.

The results of the kinetic analysis with 2TCM (Fig 5) indicated small differences between the two SA values for constants *K*_*1*_ and *k*_*2*_, but relatively large differences for the constants *k*_*3*_ and *k*_*4*_. In particular, a significant difference in *BP*_*ND*_ (*k*_*3*_/*k*_*4*_) was observed between the two SAs in the cerebellum, thalamus, hippocampus, striatum, cingulate cortex and substantia nigra. Similarly, *V*_*T*_ values in the cerebellum, thalamus, striatum and substantia nigra were detected with significant differences between the two SAs. These results demonstrate remarkable differences in the specific binding for mGluR1 between the two SA values.

When [^11^C]ITMM with conventional SA containing 355–732 pmol of nonradioactive carrier was administered, about 4–7 pmol/mL of nonradioactive carrier was estimated to reach the brain. Meanwhile, contamination by nonradioactive carrier in the administration of [^11^C]ITMM with ultra-high SA would be negligible (6–17 pmol). Previously, densities (B_max_) of mGluR1 in the rat brain have been reported as follows: 36 in the thalamus, 28 in the hippocampus, 22 in the striatum, and 20 pmol/mL in the cingulate cortex [27]. It can be assumed that 10–20% of specific binding in PET images using [^11^C]ITMM with conventional SA was occupied by nonradioactive carrier. Increases in the *BP*_*ND*_ and specific binding for mGluR1 of [^11^C]ITMM with ultra-high SA were due to a reduction in the level of nonradioactive carrier. However, in the cerebellum, the mGluR1-richest region, significant increment of specific binding of [^11^C]ITMM with ultra-high SA was detected. In our previous unpublished data, in vitro binding assay with [^18^F]FITM, an analogue of [^11^C]ITMM, using cerebellum homogenate has exhibited two binding sites of [^18^F]FITM possessing high and low affinities (S4 Fig). Hence, increments of binding of [^11^C]ITMM with ultra-high SA in the cerebellum would be caused by decrease of carrier exposure in a binding site with low affinity.

The *V*_*T*_-scaled parametric PET images of [^11^C]ITMM with ultra-high SA significantly improved visualization of mGluR1 in mGluR1-moderate or-low regions compared with the conventional SA (Fig 6). These images showed a significant improvement in the specific binding for mGluR1 using [^11^C]ITMM with ultra-high SA.

We found significant differences between the two SAs in the basal ganglia, such as striatum, thalamus, and substantia nigra. In previous studies, the B_max_ of mGluR1 in the human cerebral cortex was 26 pmol/mL, but has not been reported for the striatum [11]. The radioactive uptake (SUV) of [^11^C]ITMM with conventional SA in the human striatum is half that of the cerebral cortex [18]. This result suggests that the B_max_ in human striatum would at most be less than half of 26 pmol/mL. In a first-in human PET study using [^11^C]ITMM, 0.001–0.004% of the injection dose/mL of [^11^C]ITMM containing 5.5–12 nmol nonradioactive carrier reached the brains of healthy humans [18] This suggests that 0.1–0.5 pmol/mL nonradioactive carrier would occupy the binding sites of mGluR1 in human brains, which is lower by one order than rodents. Although the influence on receptor occupancy by nonradioactive carrier in human subjects is small, radioactive uptake in very low-density regions would be affected. In fact, radiolabeling techniques with ultra-high SA has enabled observation of slight differences in regions with very low density of the target receptor (0.7–2.3 pmol/mL for extrastriatal dopamine D_2_ receptors) [33]. Hence, PET studies using [^11^C]ITMM with ultra-high SA may permit detection of significant regional differences in mGluR1 in the basal ganglia of humans.

Interestingly, mGluR1 pathology in neurodegenerative disorders is beginning to be understood using experimental models in rodents and nonhuman primates. In a mouse model of Huntington’s disease, in which there is reduced binding of [^3^H]dopamine in the striatum, a significant decrease in mGluR1 but not mGluR5 was detected in the basal ganglia [38]. Furthermore, decreased striatal mGluR1 was reported in a rat model of ischemic stroke [39]. In nonhuman primates, mGluR1 expression is decreased in the globus pallidus and substantia nigra in Parkinson’s disease [40]. Although clinical studies in humans are very important, investigating mGluR1 in experimental animal models can help further understanding of the interactions between dysfunction of mGluR1 and related pathologies. Therefore, improving the specific binding of [^11^C]ITMM with ultra-high SA for mGluR1 will improve visualization in the rat brain, allowing clear detection of early mGluR1 changes in experimental models of brain diseases.

## Conclusions

[^11^C]ITMM with ultra-high SA demonstrated improved specific binding for mGluR1 in several brain regions, particularly in the thalamus, striatum, and substantia nigra. In vivo monitoring of mGluR1 by PET using [^11^C]ITMM with ultra-high SA can further our understanding of unknown mechanisms of mGluR1, and may help in the development of new medications for CNS disorders, such as schizophrenia.

## Supporting Information

S1 Fig Representative PET/MRI fused images of [^11^C]ITMM with (A) conventional SA and (B) ultra-high SA in the cingulate cortex (Ci)/striatum (Str), hippocampus (Hi)/thalamus (Th), and cerebellum (Ce)/pons (Po) coronal slices. The colored scale bar represents levels of radioactive uptake (standardized uptake value, SUV).
(TIF)

S2 Fig Theoretical plasma input Cp) and tissue time-activity (Ct) curves of [^11^C]ITMM with conventional SA in the cerebellum (A), thalamus (B), hippocampus (C), striatum (D), cingulate cortex (E), substantia nigra (F), and pons (G). Non-linear regressions were performed with three-term exponential curve fitting.
(TIF)

S3 Fig Theoretical plasma input (Cp) and tissue time-activity (Ct) curves of [^11^C]ITMM with ultra-high SA in the cerebellum (A), thalamus (B), hippocampus (C), striatum (D), cingulate cortex (E), substantia nigra (F), and pons (G). Non-linear regressions were performed by three-term exponential curve fitting.
(TIF)

S4 Fig Scatter plots analysis on in vitro binding assay with [^18^F]FITM using cerebellum homogenate. (TIF)
