# Supplementary figures and images for: Improved Visualization and Specific Binding for Metabotropic Glutamate Receptor Subtype 1 (mGluR1) Using [11C]ITMM with Ultra-High Specific Activity in Small-Animal PET
Source: PLoS One. 2015 Jun 15;10(6):e0130006. doi: 10.1371/journal.pone.0130006 (PMC4468202; doi:10.1371/journal.pone.0130006)

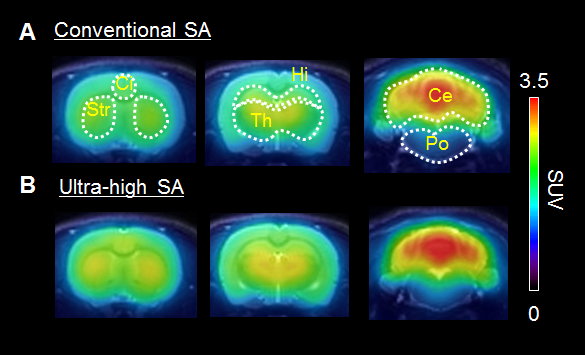

Supplement: S1 Fig — The colored scale bar represents levels of radioactive uptake (standardized uptake value, SUV). (TIF) [file pone.0130006.s001.tif]

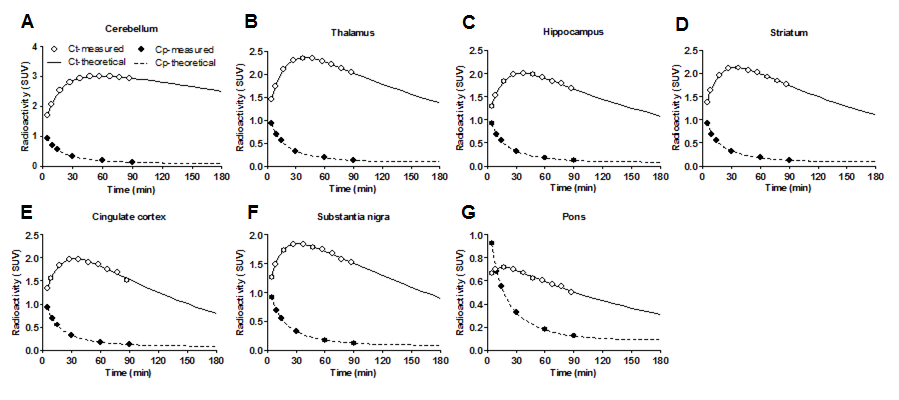

Supplement: S2 Fig — Non-linear regressions were performed with three-term exponential curve fitting. (TIF) [file pone.0130006.s002.tif]

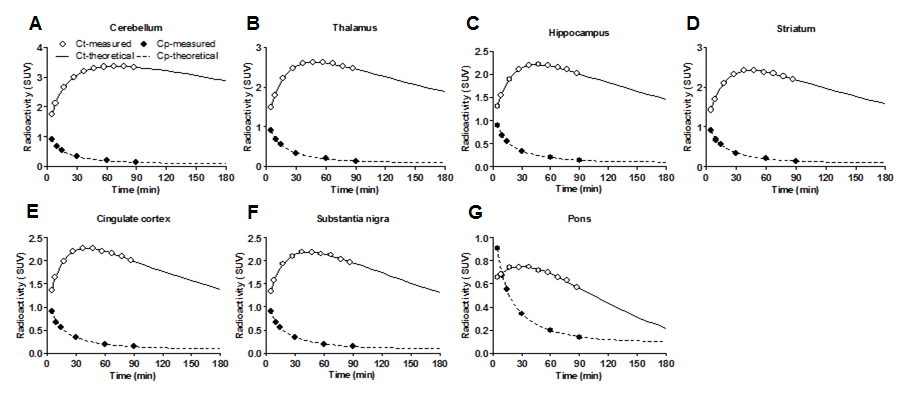

Supplement: S3 Fig — Non-linear regressions were performed by three-term exponential curve fitting. (TIF) [file pone.0130006.s003.tif]

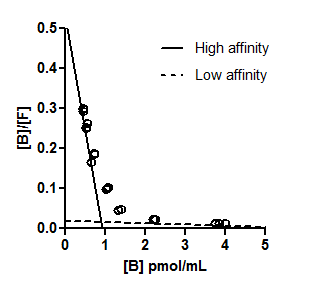

Supplement: S4 Fig — (TIF) [file pone.0130006.s004.tif]
